# Prediction of factors influencing the timing and prognosis of early tracheostomy in patients with multiple rib fractures: A propensity score matching analysis

**DOI:** 10.3389/fsurg.2022.944971

**Published:** 2022-09-23

**Authors:** Bing Zhang, Gong-Ke Li, Yu-Rong Wang, Fei Wu, Su-Qin Shi, Xin Hang, Qin-Ling Feng, Yong Li, Xian-Yao Wan

**Affiliations:** ^1^ Department of Critical Care Medicine, The First Affiliated Hospital of Dalian Medical University, Dalian, China; ^2^ Department of Emergency Intensive Care Medicine(EICU), affiliated Hospital of Yangzhou University, Yangzhou, China; ^3^ Department of Critical Care Medicine, affiliated Hospital of Yangzhou University, Yangzhou, China

**Keywords:** early tracheostomy, multiple rib fractures, propensity score matching, survival, a propensity score matching analysis

## Abstract

**Objective:**

To investigate the factors affecting the timing and prognosis of early tracheostomy in multiple rib fracture patients.

**Methods:**

A retrospective case-control study was used to analyze the clinical data of 222 patients with multiple rib fractures who underwent tracheotomy in the Affiliated Hospital of Yangzhou University from February 2015 to October 2021. According to the time from tracheal intubation to tracheostomy after admission, the patients were divided into two groups: the early tracheostomy group (within 7 days after tracheal intubation, ET) and late tracheostomy group (after the 7th day, LT). Propensity score matching (PSM) was used to eliminate the differences in baseline characteristics Logistic regression was used to predict the independent risk factors for early tracheostomy. Kaplan–Meier and Cox survival analyses were used to analyze the influencing factors of the 28-day survival.

**Results:**

According to the propensity score matching analysis, a total of 174 patients were finally included in the study. Among them, there were 87 patients in the ET group and 87 patients in the LT group. After propensity score matching, Number of total rib fractures (NTRF) (*P* < 0.001), Acute respiratory distress syndrome (ARDS) (*P* < 0.001) and Volume of pulmonary contusion(VPC) (*P* < 0.000) in the ET group were higher than those in the LT group. Univariate analysis showed that the patients who underwent ET had a higher survival rate than those who underwent LT (*P* = 0.021). Pearson's analysis showed that there was a significant correlation between NTRF and VPC (*r* = 0.369, *P* = 0.001). A receiver operating characteristic(ROC)curve analysis showed that the areas under the curves were 0.832 and 0.804. The best cutoff-value values of the VPC and NTRF were 23.9 and 8.5, respectively. The Cox survival analysis showed that the timing of tracheostomy (HR = 2.51 95% CI, 1.12–5.57, *P* = 0.004) and age (HR = 1.53 95% CI, 1.00–2.05, *P* = 0.042) of the patients had a significant impact on the 28-day survival of patients with multiple rib fractures. In addition, The Kaplan–Meier survival analysis showed that the 28-day survival of patients in the ET group was significantly better than that of the LT group, *P* = 0.01.

**Conclusions:**

NTRF, ADRS and VPC are independent risk factors for the timing and prognosis of early tracheotomy. A VPC ≥ 23.9% and/or an NTRF ≥ 8.5 could be used as predictors of ET in patients with multiple rib fractures. Predicting the timing of early tracheostomy also need prediction models in the future.

## Introduction

Multiple rib fractures (defined as the number of fractured ribs being more than 3) are usually caused by chest trauma, which might be combined with pulmonary contusion, hemothorax, pneumothorax, and potentially possibly a life-threatening lung injury (1). Patients with multiple rib fractures might require a tracheostomy because of severe injury to the respiratory system. It was reported that the incidence of tracheostomy in critically injured patients with acute respiratory failure was up to 59.0% (2).

Prompt implementation of early tracheotomy (ET) may benefit patients with multiple rib fractures. These benefits include shortening the time of sedation (3), reducing the time of ventilator use, shortening the ICU length of stay (ICULOS) and hospital length of stay (HLOS) (4). In addition, patients undergoing ET surgery can reduce the incidence of pneumonia and short-term mortality (3–6). However, tracheostomy can also cause complications such as bleeding, tracheal stenosis, and incisional skin ulcers (7, 8). There are few studies on the timing and prognostic factors of early tracheotomy. Some single-center and retrospective studies have shown that. A severe Glasgow Coma Scale (GCS) score (≤8), flail chest, and Injury Severity Score (ISS) might be risk factors that might cause patients with multiple rib fractures to have a prolonged mechanical ventilation time (9). Other researchers have also found that the clinical application of tracheostomy predictive models is limited because of a poor predictive sensitivity and a positive predictive value of only 30%–45% (10). Therefore, how to predict the timing and prognosis of ET is a difficult problem for clinicians.

Therefore, we hypothesized that there were some clinical indicators that could influence the timing and prognosis of early tracheostomy. To find suitable indicators to predict the timing and prognosis of early tracheostomy in patients with multiple rib fractures, our study used the propensity score matching analysis to verify the general data and clinical results of the patients.

## Material and methods

### Participants

**Figure 1:**
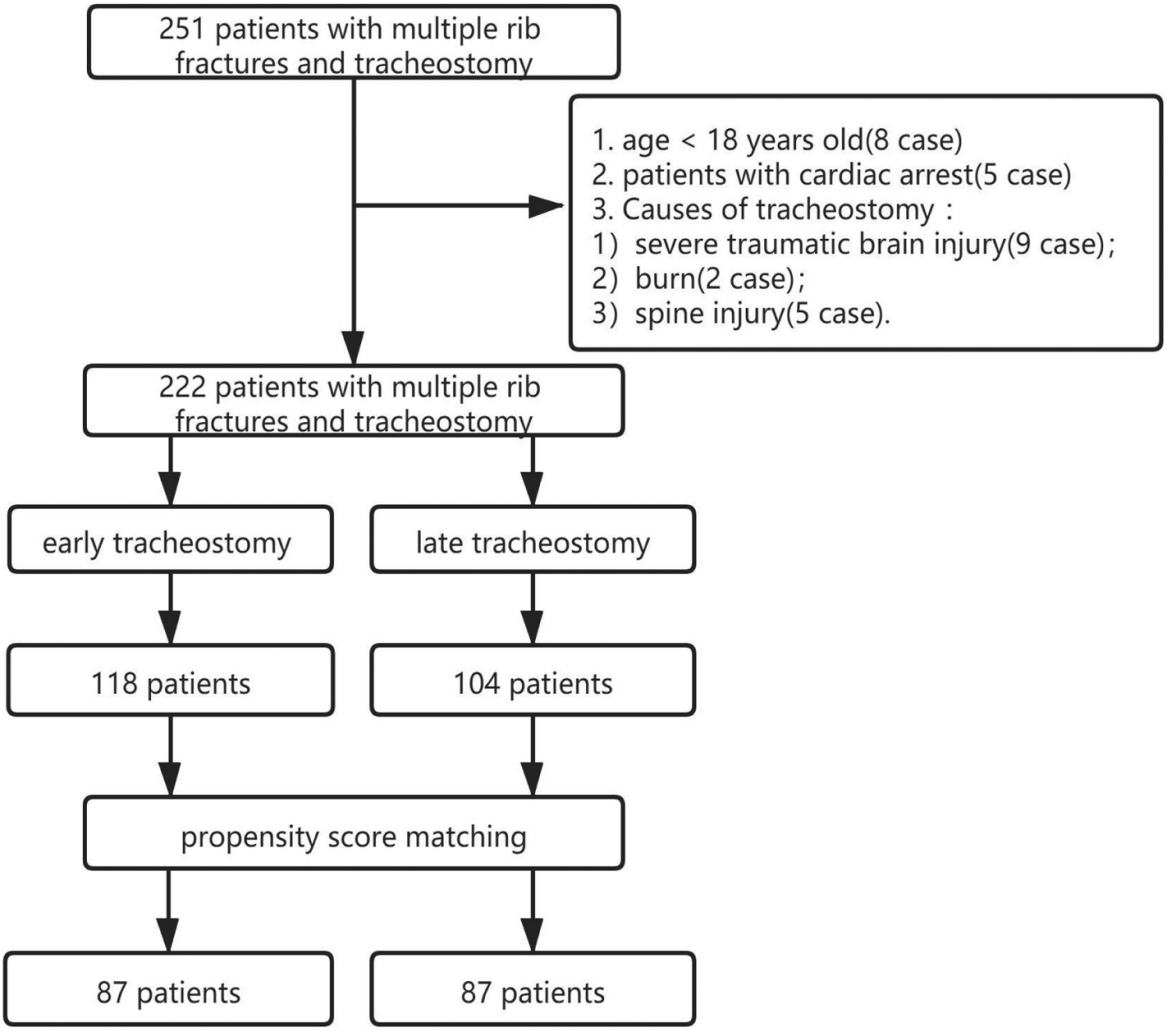
Flow chart of the study design and the patient selection.

A total of 251 patients with multiple rib fractures who underwent a percutaneous tracheostomy and who were radiologically confirmed to have multiple rib fractures (computed tomography scans, CTs) in the ICU of the affiliated hospital of Yangzhou University from February 2015 to October 2021 were retrospectively analyzed. The pretreatment evaluation included complete history and laboratory tests of patients. The inclusion criteria were as follows: (1) age >18 years; (2) multiple rib fractures diagnosed by computer tomography; (3) chest injury; (4) tracheostomy treatment after admission. We excluded patients who were <18 years old, who had cardiac arrest, and who underwent tracheostomy due to a severe traumatic brain injury, burns, or a spinal injury. According to the 2009 tracheostomy timing management guide (11), the patients in this study were divided into two groups. early tracheotomy (ET) was defined as a tracheostomy within 7 days after tracheal intubation, and Late tracheostomy (LT) was defined as a tracheostomy after the 7th day. The flowchart of patient enrollment is shown in Figure 1. Approval was obtained from the institutional review board [Ethics approval number: 2020-YKL12-23-(01)]. Informed consent was waived because of the retrospective nature of this study.

### Data collection

**Figure 2:**
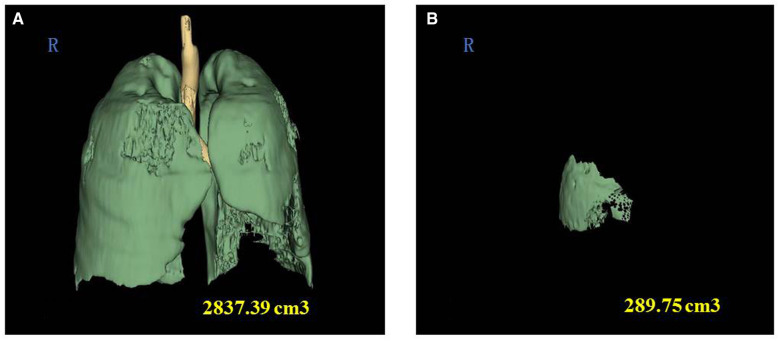
Three-dimensional reconstruction of chest CT images to calculate the volume fraction of pulmonary contusion. (**A**) Three-dimensional reconstruction of chest CT images showing the total volume of pulmonary contusion. (**B**) Three-dimensional reconstruction of chest CT images of a 61-year-old female patient showing the volume of pulmonary contusion. The volume fraction of pulmonary contusion = 289.75 cm^3^/2,837.39 cm^3^ *100% = 10.2%.

The data for this study were extracted independently by two blinded nurses. The patient's basic information were extracted from the inpatient registries and the patient electronic medical records. The indicators of the data to be extracted in this study are shown in Tables 1, 2. To measure the contusion volumes, we documented and reconstructed each of the admission chest CTs in a 3D image by computer software (Advantage Workstation 4.5, GE Healthcare) (Figure 2). We also reconstructed the pulmonary fields bilaterally and measured the pulmonary volume. The total PC volume = the volume of PC in both pulmonary fields/total pulmonary volume ∗ 100%. We defined ARDS according to the Berlin definition (12).

**Table 1:** Comparison of baseline data of patients before and after propensity score matching.

Variables	Before propensity matching			After propensity matching		
	ET (*N* = 118)	LT (*N* = 104)	*P*-value	ET (*N* = 87)	LT (*N* = 87)	*P*-value
Age, years, points	50.5 ± 15.6	47.5 ± 18.2	0.196	48.2 ± 16.1	47.6 ± 20	0.818
Male sex, *n* (%)	84 (71.1)	76 (73.1)	0.754	67 (77.0)	59 (67.8)	0.175
GCS at admission, points	9.1 ± 4.3	9.0 ± 4.5	0.893	9.2 ± 4.3	8.9 ± 4.6	0.660
ISS, points	38.9 ± 7.2	40.1 ± 9.4	0.294	39.3 ± 7.8	40.0 ± 8.8	0.580
Cardiovascular diseases, *n* (%)	21 (17.8)	8 (7.7)	0.026	13 (14.9)	8 (9.2)	0.245
Lung disease, *n* (%)	12 (10.2)	12 (11.5)	0.743	9 (10.3)	10 (11.5)	0.808
Traumatic Brain Injury, *n* (%)	84 (71.2)	72 (69.2)	0.750	60 (69.0)	62 (71.3)	0.740
Acute Respiratory Distress Syndrome, *n* (%)	76 (64.4)	48 (46.2)	0.006	59 (67.8)	37 (42.5)	0.001
Volume of pulmonary contusion, points	21.1 ± 18.0	5.9 ± 8.7	0.000	33.8 ± 11.4	20.1 ± 9.8	0.000
Number of ribs fractured, points	5.8 ± 2.1	6.1 ± 2.1	0.218	5.7 ± 2.1	6.2 ± 2.1	0.167
Number of total rib fractures, points	9.1 ± 2.4	7.9 ± 2.2	0.001	10.8 ± 2.7	7.9 ± 2.0	0.001
First rib fractures, *n* (%)	40 (33.9)	34 (32.7)	0.849	30 (34.5)	29 (33.3)	0.873
Sternum fractures, *n* (%)	37 (31.4)	31 (29.8)	0.803	26 (29.9)	24 (27.6)	0.738
Flail chest, *n* (%)	20 (16.9)	24 (23.1)	0.253	14 (16.1)	22 (25.3)	0.134
Spine coinjuries, *n* (%)	12 (10.2)	4 (3.8)	0.069	10 (11.5)	4 (4.6)	0.094
Maxillofacial coinjuries, *n* (%)	8 (6.9)	12 (11.5)	0.232	8 (9.4)	9 (10.3)	0.838
Initial value of blood lactate, mmol/L	4.0 ± 2.9	4.1 ± 2.2	0.836	4.1 ± 2.9	3.8 ± 2.1	0.509
Hemothorax, *n* (%)	58 (49.2)	32 (32.0)	0.010	41 (47.1)	31 (35.6)	0.124
Pneumothorax, *n* (%)	70 (59.3)	56 (53.8)	0.411	50 (57.5)	51 (58.6)	0.878
Initial value of hemoglobin,g/dl	11.4 ± 2.1	10.4 ± 3.4	0.424	10.9 ± 3.1	11.2 ± 2.7	0.064
Maximum value of hemoglobin,g/dl	14.6 ± 4.4	13.4 ± 3.4	0.075	14.8 ± 4.5	12.9 ± 4.6	0.092
Minimum value of hemoglobin,g/dl	8.4 ± 2.3	7.9 ± 2.4	0.325	8.7 ± 3.2	8.2 ± 2.5	0.081
Total amount of RBC transfusion,L	0.8 (0.5,1.1)	0.8 (0.6,1.3)	0.236	0.7 (0.4,1.2)	0.8 (0.5,1.1)	0.055
Total amount of plasma transfusion,L	0.9 (0.4,1.4)	0.9 (0.5,1.2)	0.465	0.9 (0.6,1.5)	0.8 (0.3,1.1)	0.056
Total amount of platelets transfusion,L	0.7 (0.3,1.2)	0.6 (0.4,1.1)	0.435	0.7 (0.3,1.1)	0.6 (0.3,1.0)	0.711
Dopamine use duration,hour,points	17.2 ± 4.5	17.0 ± 4.1	0.552	16.9 ± 4.0	17.0 ± 3.9	0.112
Noradrenaline use duration,hour,points	21.1 ± 6.8	20.9 ± 6.5	0.512	19.9 ± 4.3	21.0 ± 6.4	0.072
Vasopressin use duration,hour,points	18.2 ± 5.5	18.1 ± 5.0	0.771	18.0 ± 5.9	17.9 ± 5.8	0.091
Bleeding control procedure, *n* (%)	10 (8.5)	9 (8.7)	0.064	8 (9.2)	8 (9.2)	0.121
Surgery on limbs and joints, *n* (%)	6 (5.1)	4 (3.8)	0.073	3 (3.4)	2 (2.3)	0.502
Surgery on pelvic, *n* (%)	1 (0.8)	3 (2.6)	0.124	1 (1.1)	2 (2.2)	0.773
Surgery on abdominal, *n* (%)	1 (0.8)	2 (1.9)	0.064	1 (1.1)	2 (2.2)	0.812
Timing of tracheostomy, day, points	4.0 ± 1.3	12.6 ± 3.1	0.000	4.1 ± 1.3	12.5 ± 3.0	0.001

ET, early tracheostomy; LT, late tracheostomy; GCS, glasgow coma scale; ISS, injury severity score; RBC, red blood cell.

**Table 2:** Comparison of clinical outcomes of patients before and after propensity score matching.

Outcomes	Before propensity matching			After propensity matching		
	ET (*N* = 118)	LT (*N* = 104)	*P*-value	ET (*N* = 87)	LT (*N* = 87)	*P*-value
Duration Of mechanical ventilation, days	13.5 ± 4.1	15.7 ± 5.5	0.078	13.9 ± 3.0	15.9 ± 4.7	0.190
Duration of tracheostomy, days	27.3 ± 6.5	35.8 ± 8.2	0.180	28.4 ± 6.1	39.4 ± 7.1	0.143
Hospital lenght of stay, days	18.3 ± 6.0	17.7 ± 5.1	0.855	19.3 ± 6.6	17.0 ± 5.8	0.521
ICU lenght of stay, days	7.1 ± 2.7	11.5 ± 3.6	0.145	8.7 ± 2.1	11.6 ± 2.7	0.412
Thoracic close drainage, *n* (%)	66 (55.9)	44 (42.3)	0.043	46 (52.9)	39 (44.8)	0.288
Number of fiber bronchoscope use, points	2.5 ± 5.4	1.7 ± 2.9	0.194	2.8 ± 5.9	1.7 ± 2.8	0.129
Multidrug resistance bacteria, *n* (%)	36 (30.5)	34 (32.6)	0.335	29 (33.3)	28 (32.2)	0.732
Ventilator associated pneumonia, *n* (%)	24 (20.3)	20 (19.2)	0.657	18 (20.7)	19 (21.8)	0.732
Antibiotic use, days	10.0 ± 3.4	9.2 ± 2.6	0.722	13.5 ± 4.3	14.1 ± 4.6	0.876
Sedatives and analgesics use, days	11.4 ± 9.1	13.7 ± 8.6	0.060	11.6 ± 9.2	13.5 ± 8.3	0.151
28-day mortality, *n* (%)	24 (20.3)	29 (27.9)	0.015	19 (21.8)	23 (26.4)	0.021

ICU, intensive care unit; ET, early tracheostomy; LT, late tracheostomy.

#### Treatment

All patients received standard treatments: analgesia and sedation, bronchoscopy and alveolar lavage, and chest physical therapy. (1) The analgesia and sedation both included the use of systemic drugs, and our purpose was to enable patients to breathe effectively, promote the clearance of airway secretions and control the development of atelectasis. (2) Bronchoscopy and alveolar lavage could further optimize the patients' airway environment and could prevention pneumonia in the patients. (3) The chest physical therapy included sputum suction, artificial airway management (turning over and buckling of the back to promote sputum drainage), mechanical sputum-assisted removal of airway secretions, chest belt external fixation, closed thoracic drainage, nasal or oral endotracheal intubation to establish an artificial positive airway pressure and tracheostomy treatment. The tracheostomies were performed according to the classic indications (13). All operations were performed by physicians with extensive clinical experience. The specific methods were carried out in accordance with the latest guidelines (14).

### Statistical analysis

IBM SPSS software version 22.0 (IBM, Armonk, NY) was used for the statistical analysis. Continuous variables and normally distributed data are presented as mean ± SD, whereas categorical variables are presented as cases (n) and percentage rate (%). Continuous nonnormally distributed variables are presented as median with interquartile range (IQR) Propensity score matching was performed to eliminate the differences in baseline characteristics between the LT group and the ET group. We included the following covariates: age, ISS, GCS, and timing of tracheostomy. Nearest neighbor matching (1:1) was used, with a caliper width equal to 0.2 of the standard deviation. Logistic regression was used to predict the independent risk factors for early tracheostomy, and a Pearson correlation analysis was performed on the independent factors that were generated. ROC curves were used to compare the significance of the risk factors affecting early tracheostomy. Kaplan–Meier and Cox survival analyses were used to analyze the influencing factors of the 28-day survival. The differences with a *P* value <0.05 were significant.

## Results

### Characteristics of the patients

A total of 251 patients who had a tracheostomy were evaluated, of which 29 were excluded [8 patients aged <18 years old, 5 patients with cardiac arrest, 9 patients with a tracheostomy due to severe traumatic brain injury, 2 patients with burn injuries, and 5 patients with spinal injuries]. Finally, 222 patients met the enrollment criteria. The average age of the patients was 50 years old, and there were 160 males (72.1%). The most common injuries were traffic accident injuries (156 cases, 70.3%), followed by high fall injuries (41 cases, 18.5%), fall injuries (12 cases, 5.4%), crush injuries (11 cases, 4.9%), and unexplained injuries (2 cases, 0.9%). Among the patients who were eventually enrolled in the study, 118 patients (53.2%) were included in the ET group, and 104 patients (46.8%) were included in the LT group. On account of the propensity score matching analysis, there were 87 patients in the ET group and 87 patients in the LT group. Hence, a total of 174 patients were sampled for the final analysis (Figure 1).

Before propensity score matching, significant differences in cardiovascular disease, ARDS, the VPC, the NTRF, and hemothorax were observed between the ET and LT groups (*P* < 0.05). After propensity score matching, ARDS, the VPC, and the NTRF were significantly different. Nevertheless, age, sex, GCS, the ISS, lung disease, TBI, number of fractured ribs, first rib fracture, combined injury (flail chest, maxillofacial, spine, hemothorax, pneumothorax), initial value of blood lactate, initial value of hemoglobin, maximum value of hemoglobin, minimum value of hemoglobin, total amount of Blood transfusion, bleeding control procedure, vasopressor use duration, and surgery(on limbs and joints, pelvic, and abdominal) were not significantly different between the ET group and LT group (*P* > 0.05). In addition, the timing of tracheostomy in the patients in the ET and LT groups were (4.1 ± 1.3) days vs. (12.5 ± 3.0) days, which were significantly different (*P* < 0.05) (Table 1).

#### Comparison of patient outcomes

Before propensity score matching, the ratio of patients having closed thoracic drainage and fungal infections in the ET group was higher than that in the LT group (*P* < 0.05), while there was no significant difference after matching (*P* > 0.05). The 28-day mortality of the ET group was lower than that of the LT group, and the difference is statistically significant (*P* < 0.05) (Table 2).

**Figure 3:**
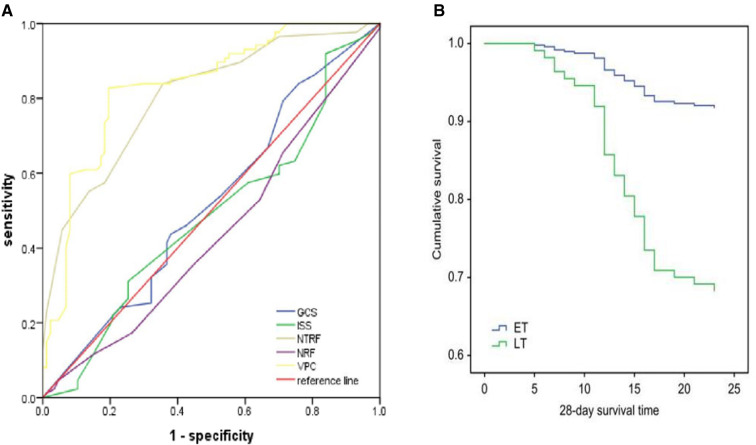
Results of the ROC curve analysis (**A**). Kaplan–Meier survival analysis based on the 28-day mortality (**B**). GCS, Glasgow coma score; ISS, injury severity score; ARDS, acute respiratory distress syndrome; VPC, volume of pulmonary contusion; NRF, number of rib fractures; NTRF, number of total rib fractures. ET, early tracheostomy; LT, late tracheostomy.

The Cox survival analysis showed that the timing of tracheostomy (HR = 2.51 95% CI, 1.12–5.57, *P* = 0.004) and age (HR = 1.53 95% CI, 1.00–2.05, *P* = 0.042) of the patients had a significant impact on the 28-day survival of patients with multiple rib fractures (Table 3). In addition, The Kaplan–Meier survival analysis showed that the 28-day survival of patients in the ET group was significantly better than that of the LT group, *P* = 0.01 (Figure 3B).

**Table 3:** COX survival analysis based 28-day mortality.

Variables	*HR*	95%*CI*	*P-*value
gender	0.87	0.44–1.72	0.058
age	1.53	1.00–2.05	0.042
cardiovascular disease	0.84	0.79–2.40	0.424
GCS	0.91	0.83–1.00	0.085
ISS	0.99	0.95–1.04	0.054
ARDS	1.64	0.78–3.45	0.073
VPC	0.99	0.97–1.02	0.062
NTRF	1.02	0.90–2.16	0.081
timing of tracheostomy	2.51	1.12–5.57	0.004

*HR,* hazard ratio; 95%*CI,* 95% confidence interval; GCS, glasgow coma scale; ISS, injury severity score; ARDS, acute respiratory distress syndrome; VPC, volume of pulmonary contusion; NTRF, number of total fractures of the ribs.

### Analysis of independent risk factors for early tracheostomy

**Figure 4:**
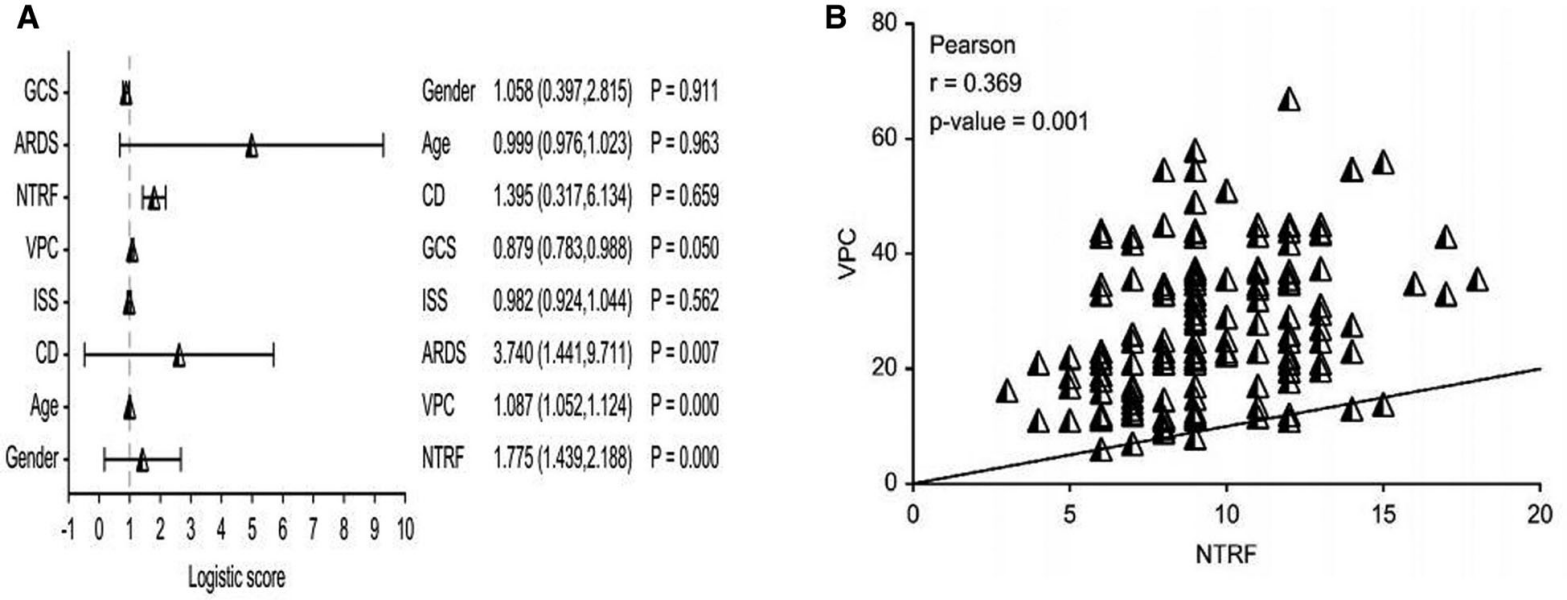
Results of the multivariate logistic regression analysis of the patients (**A**). The correlation analysis results of the volume of pulmonary contusion and the total number of fractures of the ribs (**B**). CD, cardiovascular diseases; GCS, Glasgow coma score; ISS, injury severity score; ARDS, acute respiratory distress syndrome; VPC, volume of pulmonary contusion; NTRF, number of total rib fractures.

The results of multivariate logistic regression analysis showed that ARDS (*P* = 0.007), the VPC (*P* = 0.000) and the NTRF (*P* = 0.000) were three independent risk factors for early tracheostomy in patients with multiple rib fractures(Figure 4A). The Pearson correlation analysis of the three independent factors showed that Moderate correlation between VPC and NTRF (*R* = 0.369, *P* = 0.001) and the relationship between ARDS and VPC were weakly related (*R* = 0.179, *P* = 0.018)., *R* = 0.179, *P* = 0.018; and Moreover, the NTRF and ARDS were not obviously correlated (*R* = 0.132, *P *= 0.110) (Figure 4B).

### Analysis of prognostic factors of early tracheostomy

The ROC curve analysis showed that the areas under the curve of the VPC and NTRF were 0.804 and 0.832, respectively, *P* = 0.001. The GCS, ISS and NFR scores were not significantly different between the groups (*P* > 0.05) (Figure 3A). We calculated that the maximum value of the VPC Youden index [sensitivity—(1-specificity)] was 0.633, corresponding to a sensitivity = 0.828, and the corresponding cutoff-value of the VPC was 23.9. Additionally, we also calculated that the maximum value of the NTRF Youden index was 0.474, the corresponding sensitivity was 0.839, and the corresponding cutoff-value of the NTRF was 8.5.

## Discussion

Many researchers have tried to predict the influencing factors of early tracheostomy in ICU patients. Most of these studies have focused on specific subgroups, such as patients with multiple injuries (15–18), spontaneous cerebral hemorrhage (19, 20), and hypoxic/hypoxic-ischemic encephalopathy (21). However, there is a lack of large-scale observational studies on patients with chest trauma, especially in patients with multiple rib fractures. Multiple rib fractures and flail chest have high mortality (18.7%) due to the associated complications of acute respiratory distress syndrome, pneumonia and haemorrhage. The research on the timing of tracheotomy and related factors of prognosis has important clinical significance for the treatment of patients with multiple rib fractures.

Several retrospective studies have shown that severe brain injuries, flail chest, a severe thoracic trauma score, lung contusion, and rib fractures (22, 23) were risk factors for receiving mechanical ventilation more than 7 days in patients with multiple rib fracture. Nevertheless, our study excluded patients who were in a coma for longer than 72 h due to a TBI to reduce the impact of tracheostomy in patients with a prolonged coma. In addition, we counted the NTRF in each enrolled patient, which quantified the severity of the chest injury more than a flail chest injury did. Fokin et al. (24) found that the timing of tracheostomy was not affected regardless of the total number of rib fractures ≥5 or ≥6. However, our study found that when the NTRF was ≥8.5, patients might require early tracheostomy, and ET may reduce 28-day mortality in patients with multiple rib fractures. We speculate that the NTRF value (8.5) may be the threshold of the number of rib fractures affecting the outcome of the patient. The studies of Battle et al. (25) and Shulzhenko et al. (26) also showed the same results. In addition, previous studies have found that the VPC could quantify the severity of lung contusion (11, 27, 28). For the first time, we used the VPC as a research variable for tracheostomy in patients with multiple rib fractures. When the VPC ≥ 23.9, it indicated that the patient might need early tracheostomy, and this VPC value was also related to the patient's survival benefit. This result was consistent with the studies of Mahmood et al. (27) and Wang et al (28). Finally, we found that there was a significant correlation between the NTRF and VPC. The surgical internal fixation usually significantly reduces the HLOS, ICU LOS, and the incidence of pneumonia (29, 30). However, our study showed that the HLOS, ICU LOS, and the incidence of pneumonia were not significantly different between the two groups. The participants included in our study were patients with severe chest trauma who received mechanical ventilation. These patients usually had longer hospital stays and a higher incidence of ventilator-associated pneumonia. In our study, the 28-day mortality of patients in the ET group was lower than that in the LT group, and the results of survival analysis also verified that the 28-day survival of patients in the ET group was better than that in the LT group. This indicated that an the early tracheostomy was also related to the patient's survival benefit, which was consistent with the view of Raimondi et al. (31). On the contrary, Fokin et al. (23) and Kang et al. (32) reported that early tracheostomy did not reduce the mortality of trauma patients and the mortality was related to traumatic brain injury. The reason for this difference in mortality might be due to a selection bias or different monitoring modes. Moreover, we believe that if the patients were found to be a high-risk group for a tracheostomy, the patients might benefit from ET. Interestingly, chest closure drainage as one of the clinical outcomes was significantly higher in the ET group than in the LT group before propensity score matching. These results imply that the timing of tracheotomy does not have a significant effect on other clinical outcomes. However, there were no statistically significant differences between the two groups after propensity score matching for all clinical outcomes but 28-day survival. In our study, the covariates age, ISS, GCS, and timing of tracheostomy were treated with propensity score matching. This implies that these treated covariates are likely to be influential factors for other clinical outcomes. This is something that needs to be further explored. Finally, we emphasized an individualized treatment plan and do not recommend ET to reduce mortality.

This study has several limitations. First, it was a retrospective and observational study, which is subject to the limitations of this type of researchs. Although we believe that our study subjects can well represent the characteristics of patients with this kind of trauma, our study involved data from only one city in the country; Second, there are many complex factors affecting the mortality in critically ill patients, but only using a single therapeutic intervention might not change the patient mortality. Third, the baseline level of the patients in theET group and LT group were generally poor, and there was no difference in other prognostic indicators. This difference might have been influenced by many therapeutic factors. Finally, the indicators are not comprehensive enough. For example, the management of rib fractures was not considered. The management of rib fractures may also have an impact on outcome measures. Our next research plan will include more indicators, will optimize the patient grouping, and will strictly stratify the analysis. In the future, there is an urgent need to establish a more complete prediction model to predict the timing and prognosis of early tracheostomy in patients with multiple rib fractures.

## Conclusion

ARDS, the VPC, and the NTRF were independent risk factors for ET. A VPC ≥ 23.9% and/or an NTRF ≥ 8.5 could be used as predictors of ET in patients with multiple rib fractures. Early tracheotomy may benefit the 28-day survival of patients with multiple rib fractures. Predicting the timing of early tracheostomy also need prediction models in the future.

## Data Availability

The original contributions presented in the study are included in the article/Supplementary Material, further inquiries can be directed to the corresponding author/s.
